# Evaluation of chromocystoscopy in the diagnosis of cystitis in female donkeys

**DOI:** 10.1371/journal.pone.0202596

**Published:** 2018-08-29

**Authors:** Naglaa A. Abd El Kader, Haithem A. Farghali, Ashraf M. Abu-Seida, Noha Y. Salem, Marwa S. Khattab

**Affiliations:** 1 Department of Surgery, Anaesthesiology and Radiology, Faculty of Veterinary Medicine, Cairo University, Giza, Egypt; 2 Department of Internal Medicine and Infectious Diseases, Faculty of Veterinary Medicine, Cairo University, Giza, Egypt; 3 Department of Pathology, Faculty of Veterinary Medicine, Cairo University, Giza, Egypt; University Medical Center Utrecht, NETHERLANDS

## Abstract

Early detection of cystitis in equine is essential to improve the prognosis and outcome of therapy. However, the conventional white light endoscopy is not sufficiently accurate for this purpose. Hence, this study evaluated chromoendoscopy as a recent diagnostic tool for cystitis in female donkeys. For this purpose, 5 apparently normal donkeys (control group) and 5 female donkeys with cystitis (diseased group) were used. Physical and rectal examinations, urine analysis, white light cystoscopy, methylene blue-based chromoendoscopy and histopathology were performed in all animals. Turbid urine exhibiting an alkaline pH and a significant (*P* = .02) increase in the numbers of RBCs and WBCs was observed in the diseased group compared to the control one. In the control group, white light cystoscopy showed a smooth pale pink glistening mucosa with two openings of the ureters and visible submucosal blood vessels. During chromocystoscopy, faint bluish discoloration of the mucosal surface with clearly visible submucosal blood vessels was detectable. These findings were correlated with the histopathological findings of the biopsies collected from the urinary bladder. In the diseased group, white-light cystoscopy showed clearly visible blood vessels, mildly hyperaemic mucosa in focal or diffuse forms and small vesicle formation. Chromocystoscopy revealed dark bluish oedematous and irregular mucosa either in a focal form or a diffuse form (marbled appearance) with deeply stained submucosal blood vessels. Histopathologically, the urothelium was hyperplastic with squamous metaplasia and the lamina propria was infiltrated with few leukocytes and congested blood vessels. Small bluish dots representing the absorbed methylene blue dye were seen in the inflamed areas against the lightly stained mucosa of the bladder. Severe diffuse necrotic cystitis was also seen with bacterial aggregations on the surface. Gram’s staining revealed both gram positive bacilli and Gram positive coccobacilli. In conclusion, chromoendoscopy is a helpful tool for early diagnosis of cystitis in female donkeys and enables targeted biopsies, which improves the prognosis and outcome of therapy.

## Introduction

Infections of the urinary bladder (UB) and urethra are fairly common in equine and usually manifest in an abnormal urination pattern [1]. These infections of the urinary tract in equine are rarely of a primary nature and are instead generally subsequent to obstruction or other causes of abnormal urine flow [2]. Bacterial infections either ascending from a urinary tract infection (UTI) or descending from a renal infection and the development of septicaemia are the common causes of cystitis in equine. Moreover, cystitis may occur as a secondary infection after an injury during parturition or be caused by cystic calculi, neoplasms of the urinary bladder, or bladder paralysis secondary to neurological disorders [3].

Cystitis, which is more common in female equine than in males, is an inflammation of the urinary bladder mucosa. The common signs of cystitis in equine are dysuria, poor production of urine, unusual urine consistency and frequent painful urination [2,3].

The diagnosis of such cases depends mainly upon a case history, a rectal examination and urine sample analysis. Urine analysis was established as a cheap, rapid technique for preliminary diagnosis of urinary tract affections. In cystitis, pyuria, and hematuria are common findings [4], unfortunately, hematuria can be seen in other conditions such as; neoplasm of urinary tract, kidney infarction, coagulopathy, trauma, idiopathic kidney hemorrhage and urethral hemorrhages [5]. Though urine analysis gives a strong indication about urinary tract status but to assess the mucosal surface of urinary bladder and differentiate between various types of cystitis, a different modality is required.

Cystoscopy was advocated by many authors for its role in evaluating the degree of mucosal impairment, bladder wall [4], irritation of mucosa or masses [6].

Unlike urine analysis, endoscopy was able to differentiate between various types of cystitis [7–12]. Therefore, endoscopic evaluation of the urinary bladder has now gained wide popularity, especially for the assessment of mucosal thickness and damage in cases of cystitis in horses [7–12]. In a case of sabulous cystitis, cystoscopy was able to detect a large amount of sabulous substances inside the urinary bladder with irritation [9]. Another report dealt with bladder eversion in an Arabian horse, cystoscopy was able to detect ulceration and erosion in the bladder mucosal surface [10]. Moreover, Onmaz *et al*. [11] used cystoscopy for diagnosis of an advanced ulcerative cystitis in a mare suffered from recurrent cystitis.

However, equine urine is characterized by a high content of mucous and calcium carbonate crystals, which can hinder endoscopic visibility. To overcome this obstacle, drainage of urine and inflation of the urinary bladder with air have been applied prior to endoscopic examination in equine [12].

Moreover, there is a poor correlation between histopathologic diagnosis and white light endoscopic findings. Because of the patchy distribution of lesions within the mucosa, the sensitivity of random biopsies is low [13,14]. In recent years, chromoendoscopy as a novel use of the endoscope has become a widely used tool for enhancing the visibility of epithelial and mucosal lesions. In chromocystoscopy, pre-treatment of the UB mucosa with a mucolytic agent such as acetic acid is required. Acetic acid interacts with the glycoprotein layer which covers the mucosa and breaks the disulphide bonds resulting in a higher contrast of the surface epithelium. Then a vital stain such as methylene blue is applied to stain the affected cells. These chemical substances are advocated to enhance visibility and improve the ability to detect small changes in the epithelial and mucosal surface by enhancing the contrast of raised and deepened areas that might be missed during routine white light endoscopic examination [13,14]. Moreover, chromoendoscopy is a helpful tool for collecting of targeted ‘smarter’ biopsies being superior to random biopsies, thus reducing the time; effort and cost of histopathology. Therefore, this study hypothesized that chromoendoscopy may improve the diagnostic ability of traditional white light cystoscopy. Although chromoendoscopy has been used intraluminal in humans in the gastrointestinal tract (such as Barrett’s oesophagus and cervical cancers) and urinary bladder (UB) for the detection of neoplastic changes on the mucosal surface [15–18], the application of this technique for urinary bladder examination has yet to be evaluated. To our knowledge, there are no prior published reports of endoscopic image enhancement in the veterinary literature.

Early detection of cystitis in equine is essential to improve the prognosis and outcome of therapy. However, conventional white light endoscopy is not sufficiently accurate for this purpose. Hence, the present study evaluated chromoendoscopy as an early diagnostic tool for cystitis in female donkeys.

## Materials and methods

### Animals

The present study was approved by Cairo University Institutional Animal Care and Use Committee (CU-IACUC-II-F-48-18). This study was carried out on 10 female donkeys of 3–10 years old. The animals were kept in separate stables with proper ventilation and nutrition conditions that ensure good, clean and comfortable environment. Fresh and clean water was provided ad libitum. Ready mix ration was given with hay to all donkeys as feeding. Adequate straw bedding was used under all donkeys. These animals were divided into two groups, control group (n = 5) and diseased group (n = 5). The inclusion criteria of the diseased group included dysuria, hematuria and pyuria. All animals were treated in accordance with all institutional and international guidelines for animal use and care. All efforts were performed to minimize suffering.

Animals in the control group were selected from healthy non-pregnant female donkeys that used for teaching purposes.

### Physical and laboratory examinations

Upon admission of the diseased animals, their case histories and clinical signs were recorded.

Rectal examination of the urinary bladders of the affected donkeys was carried out. Urine samples were collected from both control and diseased groups through catheterization for urine analysis.

### Endoscopic examination

For cystoscopy of both normal and diseased animals, the female donkey was restrained and secured after sedation with Xylazine HCl (Xyla-Ject 2%^®^, ADWIA Co., S.A.E-Egypt) at a dose of 1mg/kg b.wt given intravenously.

The tail of the female donkey was wrapped using gauze and bandage tape and was then tied around the neck, to allow clear entry to the vulva and external urethral orifice.

Under complete aseptic measures, catheterization was performed to collect a urine sample for urine analysis and to evacuate the urinary bladder to facilitate cystoscopy examination.

After evacuation of the urinary bladder, the endoscope (a 9 mm-diameter flexible Eickemeyer video-endoscope, China) was directed by a sterile gloved lubricated hand under the vestibule-vaginal fold, just rostral to the caudal brim of the pelvis. Using a finger to guide and detect the external urethra orifice. Then, it was inserted into the urethra and guided through the urinary tract into the bladder. The urinary bladder was inflated with air according to the method described by Divers and Schott [12]. We next performed an endoscopic examination, beginning with a white light endoscopic examination (plain endoscopy).

Weak acetic acid (pH 2.5) was subsequently applied to the urinary bladder wall via catheter spraying (25–30 mL); after application, mucosal whitening was visible during white-light endoscopic examination. Next, we performed catheter spraying of 0.2% methylene blue (MB) dye (15–30 mL), which was deposited and allowed to spread on the mucosal wall of the urinary bladder, followed by endoscopic examination (chromocystoscopy). Biopsies (6–8 specimens) were collected after the examination from multiple sites of the UB in the control group and from suspicious areas of the UB in the diseased group as described by Boeriu, *et al*. [19]. In all animals, urinary bladder biopsies were picked up by a 8.5 F flexible wire biopsy forceps.

### Histopathological examination

The collected specimens obtained through biopsy were fixed in 10% neutral formalin buffer and then processed using a paraffin embedding technique. Tissue sections of 3–5 μm in thickness were obtained using a microtome (Leica 2135) and were then stained with haematoxylin and eosin as described by Suvarna *et al*. [20]. The tissue sections were finally examined via light microscopy, and lesions were photographed using an Olympus XC30 camera (Tokyo, Japan). Gram’s stain was carried out in tissue sections of urinary bladder to demonstrate Gram negative and Gram positive bacterial aggregations in the examined tissue [20].

### Treatment

All diseased animals were treated with a long course (21–28 days according to the severity of the case) of broad-spectrum antibiotics and anti-inflammatory drugs. The affected animals were given intra-muscular phenylbutazone (Phenyloject^®^, ADWIA Co., Egypt) once daily at a dose of 4 mg/kg b.wt and intra-muscular streptomycin-penicillin (Streptopenicid^®^, CID Co., Egypt) once daily at a dose of 20,000 IU/kg b.wt of penicillin and 12.5mg/kg b.wt of streptomycin.

### Statistical analysis

Data were analyzed using statistical software program (SPSS) and presented as mean ± standard deviation (SD). Analysis of variance of the collected data was performed. Significance level was set at *P* ≤ .05%.

## Results

### Clinical and laboratory findings

The most frequently recorded clinical signs were depression and an increased frequency of urination accompanied by pain, straining and discomfort. Upon urinalysis, turbid urine exhibiting an alkaline pH was reported. Also, a significant (*P* = .02) increase in the numbers of RBCs and WBCs was observed in the diseased group compared to the control one. The results of the urinalysis are shown in Table 1.

**Table 1:** Urinalysis findings in both normal and diseased female donkeys.

Parameter	Diseased female donkeys	Normal female donkeys
**Aspect**	Turbid	Turbid
**Specific gravity**	1.034±0.004	1.020–1.050
**RBCs/HPF**	41.67±4.41	<5 RBCs/HPF
**WBCs/HPF**	28.33±7.26	<10 WBCs/HPF
**pH**	8.067±0.145	7–9
**Glucose**	Nil	Nil
**Protein**	Traces	Traces

Rectal examination revealed normal UB in the control group and inflamed UB and pain in the diseased group.

### Cystoscopic and histopathological findings in the control group

**Fig 1:**
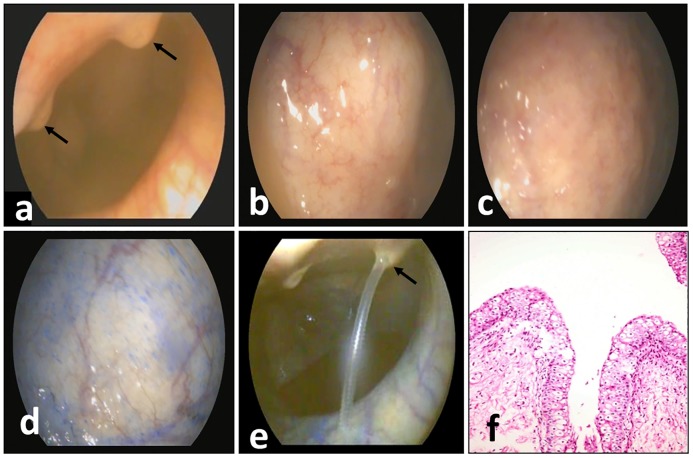
(a) Plain cystoscopy in a normal female donkey showing 2 ureteral orifices (black arrows) on the dorsal surface of the urinary bladder at 11 and 1 o’clock and pale pink mucosa with observed small blood vessels. (b) Plain cystoscopy in a normal female donkey showing a glistening smooth mucosal surface and visible submucosal blood vessels. (c) Acetic acid cystoscopy in a normal female donkey showing hyper-whitening of the mucosal surface. (d) Chromocystoscopy in a normal female donkey showing faintly discoloured mucosal surface and visible submucosal blood vessels. (e) Chromocystoscopy in a normal female donkey showing ureteral orifices with a urine stream at 1 o’clock (black arrow), normal faintly discolored mucosal surface and clearly visible submucosal blood vessels. (f) Normal histological structure of the urinary bladder in a female donkey showing intact transitional epithelium (H and E stain X 200).

In the control group, white light endoscopic examination of the UB showed a pale pink mucosa with two openings of the ureters, which appeared as two papillae entering the bladder (Fig 1a), and a small urine stream was observed once per minute. Zooming in on the mucosa, a glistening smooth mucosal surface with visible submucosal blood vessels was observed (Fig 1b).

After the spraying of acetic acid, whitening of the mucosa was visible (Fig 1c). During chromoendoscopy, faint bluish discoloration due to MB was observed (Fig 1d), and upon zooming out, faint discoloration of the mucosal surface with clearly visible submucosal blood vessels was detectable (Fig 1e).

Histopathological evaluation of the collected biopsies revealed normal urinary bladder lined by normal transitional epithelium (Fig 1f).

### Cystoscopic and histopathological findings in the diseased group

**Fig 2:**
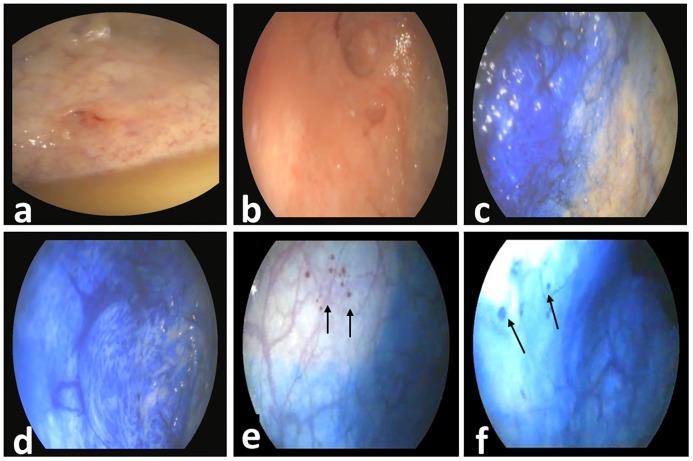
(a) Plain cystoscopy in a female donkey with cystitis showing slight hyperemic mucosa and clear submucosal blood vessels at the apex vesicae. (b) Acetic acid cystoscopy in a female donkey with cystitis showing mucosal irregularities at the apex vesicae (c) Chromocystoscopy in a female donkey with cystitis showing diffuse dark bluish discolored and edematous mucosa. (d) Chromocystoscopy in a female donkey with cystitis showing diffuse dark bluish inflamed mucosa with marbled appearance. (e&f) Chromocystoscopy in a female donkey with cystitis showing small focal dark bluish inflamed mucosal areas (arrows).

In the diseased group, white light cystoscopy showed clearly visible blood vessels, mildly hyperaemic mucosa in focal or diffuse forms and small vesicle formation at the apex vesicae (Fig 2a).

**Fig 3:**
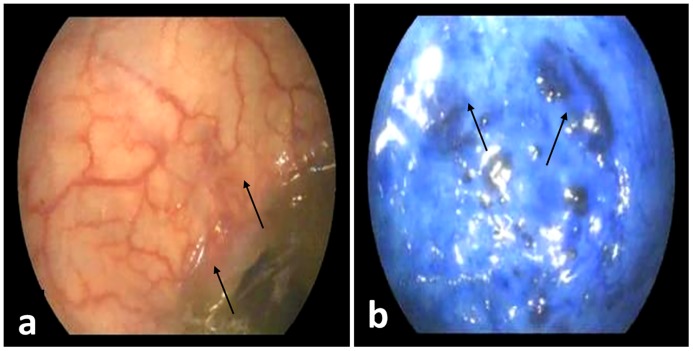
(a) Acetic acid cystoscopy in a female donkey with cystitis showing large focal edematous mucosal areas (arrows). (b) Chromocystoscopy in a female donkey with cystitis showing large deeply stained, raised mucosal areas (arrows).

Following acetic acid spraying, an irregular red mucosa was observed (Fig 2b). The inflamed areas became clearer and well defined after staining with 0.2% methylene blue dye (Fig 2c). The inflamed areas of mucosa appeared as dark bluish oedematous and irregular mucosa either in a diffuse form or focal form with deeply stained submucosal blood vessels (Fig 2c & 2d). In the diffuse form, bluish, marbled appearance of the mucosa was noticed (Fig 2d). In the focal form, the affected UB showed either small pin point dark bluish areas (Fig 2e & 2f) or large dark bluish patches (Fig 3).

**Fig 4:**
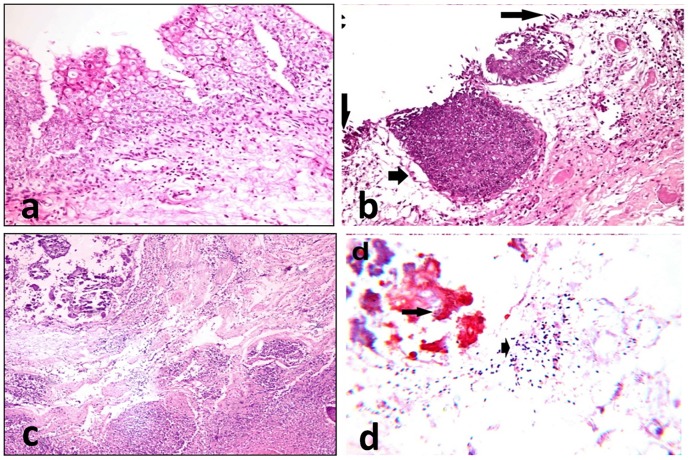
Biopsies from the urinary bladder of female donkeys with cystitis showing: (a) Focal hyperplasia of the urothelium with a few leukocytic infiltrates in the lamina propria and submucosa (Hematoxylin and eosin,×200). (b) Focal areas of erosions (long arrows) and squamous metaplasia in the urothelium (short arrow), accompanied with congested blood vessels and inflammatory cell infiltration (Hematoxylin and eosin, x200). (c) Necrosis of the the urothelium, diffuse leukocytic infiltration in the submucosa and bacterial aggregations on the surface (Hematoxylin and eosin,×100). (d) Gram-negative bacilli (long arrow) are observed on the mucosa, and Gram-positive coccobacilli (short arrow) are observed in necrosed tissue (Gram’s stain ×1000).

Histopathologically, the urothelium was hyperplastic, and the lamina propria was infiltrated with a few leukocytes (Fig 4a). In addition, squamous metaplasia in the urothelium, accompanied with congested blood vessels, inflammatory cell infiltration and focal areas of erosions was also seen (Fig 4b). Small bluish dots, against the lightly stained mucosa of the bladder were seen.

Severe diffuse necrotic cystitis was also seen, in which fibrinous exudates, necrosis with loss of urothelium and massive leukocyte infiltrates in the lamina propria and submucosa were observed (Fig 4c). Bacterial aggregations on the surface (Fig 4c) were observed. Gram’s staining revealed gram positive bacilli on the mucosa and Gram positive coccobacilli in the necrosed tissue (Fig 4d).

## Discussion

Chromoendoscopy has become a highly promoted procedure for various indications in human [21]. The present work offers a preliminary study for evaluation of chromoendoscopy as a diagnostic tool for cystitis in female donkeys. The results of this study revealed that chromocystoscopy is more helpful tool than white light cystoscopy in diagnosis of cystitis in female donkeys and an accurate tool for collecting biopsies from the affected UB. This technique is useful in predicting histological changes during the endoscopic examination itself. The obtained histopathological findings were correlated with the chromocystoscopic findings.

Similarly, chromoendoscopy has been used to detect healed gastric ulcers and improve the accuracy of the diagnosis of histological repair of gastric ulcers in humans [22–26].

The most consistent findings of urinalysis in female donkeys with cystitis were the presence of large numbers of RBCs and WBCs, along with a pH of 8.067. Similar findings were reported by Johnson *et al*. [3] in horses. Normally, equine urine contains less than 5 RBCs/HPF and less than 10 WBCs/HPF [27]. The recorded RBCS and WBCs above the reference ranges may be indicative of infection and inflammation. Similar findings were reported in geldings with ulcerative cystitis [28]. Cystoscopy is essential in cases of hematuria, presence of unusual cells in a urine sample, frequent bladder infections, or pain in the bladder, urethra or during urination. Unfortunately, white light endoscopy misses mucosal lesions, while chromoendoscopy is a diagnostic method that can detect small lesions on the mucosal surface. Chromoendoscopy has significantly enhanced the diagnostic scope of endoscopy in general [22].

In the present study, MB is sprayed onto the mucosal surface of UB to highlight the inflamed areas of UB and consequently facilitate the collection of biopsies to distinguish different types of epithelia through histopathology. Conventional white light endoscopy is characterized by a high miss rate for early gastric cancer, which is visible only as slight surface irregularities [29]. Such subtle mucosal changes are difficult to distinguish from nonspecific or inflammatory lesions.

At the beginning of chromoendoscopy, it is necessary to evacuate the urinary bladder due to the presence of normal mucoid urine, which masks visibility. Accordingly, previous findings [12] have shown that the thick mucus and dense calcium carbonate crystals preclude the use of either urine itself or an exogenous fluid to distend the bladder during cystoscopic examination, as visibility would be close to nil without arduous and extensive flushing.

In this study, we avoided over-distension of the urinary bladder, which can cause discomfort to the animal and mask the ureteral openings during cystoscopy.

During white light endoscopy, the normal urinary bladder wall is characterized by a smooth glistening mucosa and prominent submucosal blood vessels. These findings were previously recorded in horses [30].

During chromoendoscopy, the substances used for staining are generally inexpensive and readily available. These substances are classified as vital stains, such as acetic acid, MB, gentian violet and Lugol’s stain; non-absorptive contrast stains, such as indigo carmine; and reactive stains, such as Congo red and phenol red [13].

In the present study, MB had been used due to its low toxicity and low molecular weight that can easily penetrate into tissues and has been previously used in humans. In this study, MB-based chromocystoscopy was a safe technique for diagnosis of cystitis in female donkeys with limited complications, such as blue discolouration of the urine.

In this study, acetic acid was used previous to MB. It is a weak acid and is not actually a stain; however when sprayed onto the mucosa of the urinary bladder, it can enhance the structural surface pattern, similar to a contrast substance. In recent investigations, acetic acid has been sprayed over the mucosa to overcome the mucus layer covering the mucosa through interaction with the glycoprotein layer that covers the mucosa and disruption of disulphide bonds, resulting in a glistening appearance of the mucosa. These findings are parallel to those reported before [18, 19]

Biopsies were collected at the end of the endoscopic examination of the urinary bladder to avoid haemorrhage, which might hinder endoscopic visibility. Similar action had been carried out by Boeriu *et al*. [19].

In the current study, the normal bladder wall was stained a faint blue colour, which was confirmed by the histopathological results of biopsy and revealed a normal histological structure of the urinary bladder, lined by transitional epithelium. These findings are explained by how the normal urinary bladder reacts with chromoendoscopy, due to taking up a small amount of MB. This finding was obtained using MB dye, but similar findings have been recorded using other diagnostic modalities (photo-acoustic cystography) [31]. However, the inflamed areas of the urinary bladder wall appeared dark bluish. This could be explained by the large amount of MB passed through the inflamed areas and darkly stained them.

Prolonged treatment regimens of antibiotics and anti-inflammatory drugs for cystitis are essential, since relapses of infection are common due to dormant bacteria in the UB wall. The presence of various species of bacteria inside the UB of the affected female donkeys’ wall confirms the need of prolonged treatment regimens and broad spectrum antibiotics for treatment of cystitis in equine. All female donkeys were recovered after 3–4 weeks of treatment according to the severity of the case. Accordingly, bacterial cultures from urine and antimicrobial sensitivity testing of cultured bacteria are recommended to help the veterinarian to determine the antibiotic of choice and the course of treatment of cystitis in equine.

The main limitation of the present study is the small sample size. Therefore, further studies on large number of animals are recommended.

## Conclusions

Chromoendoscopy is a helpful tool for early diagnosis of cystitis in female donkeys and enables targeted biopsies, which improves the prognosis and outcome of therapy.

## Supporting information

S1 File (PDF)
